# Membrane Distillation Trial on Textile Wastewater Containing Surfactants Using Hydrophobic and Hydrophilic-Coated Polytetrafluoroethylene (PTFE) Membranes

**DOI:** 10.3390/membranes8020031

**Published:** 2018-06-15

**Authors:** Jesús Villalobos García, Noel Dow, Nicholas Milne, Jianhua Zhang, Leslie Naidoo, Stephen Gray, Mikel Duke

**Affiliations:** 1 Institute for Sustainable Industries & Liveable Cities, Victoria University, P.O. Box 14428, Melbourne, VIC 8001, Australia; Noel.Dow@vu.edu.au (N.D.); n.milne@deakin.edu.au (N.M.); Jianhua.Zhang@vu.edu.au (J.Z.); Stephen.Gray@vu.edu.au (S.G.); Mikel.Duke@vu.edu.au (M.D.); 2 Australian Textile Mills Pty Ltd., Wangaratta, VIC 3677, Australia; lnaidoo@australiantextiles.com.au; 3 Laboratoire de Génie Chimique (LGC), Université de Toulouse, CNRS, INPT, UPS, Toulouse 31432, France

**Keywords:** membrane distillation, hydrophilic membrane, polytetrafluoroethylene, desalination

## Abstract

Treating wastewater from textile plants using membrane distillation (MD) has great potential due to the high-salinity wastes and availability of waste heat. However, textile wastewaters also contain surfactants, which compromise the essential hydrophobic feature of the membrane, causing membrane wetting. To address this wetting issue, a custom-made membrane consisting of a hydrophilic layer coated on hydrophobic polytetrafluoroethylene (PTFE) was tested on textile wastewater in a pilot MD setup, and compared with a conventional hydrophobic PTFE membrane. The test was carried out with a feed temperature of 60 °C, and a permeate temperature of 45 °C. The overall salt rejection of both membranes was very high, at 99%. However, the hydrophobic membrane showed rising permeate electrical conductivity, which was attributed to wetting of the membrane. Meanwhile, the hydrophilic-coated membrane showed continually declining electrical conductivity demonstrating an intact membrane that resisted wetting from the surfactants. Despite this positive result, the coated membrane did not survive a simple sodium hydroxide clean, which would be typically applied to a membrane process. This brief study showed the viability of membrane distillation membranes on real textile wastewaters containing surfactants using hydrophilic-coated hydrophobic PTFE, but the cleaning process required for membranes needs optimization.

## 1. Introduction

The need for more sustainable industries that can capture waste and recycle clean water from their existing saline effluent drives the need for new desalination technologies. High-efficiency and low-cost thermal processes are an important part of solving some of our most important water problems. One technology that may help achieve this is membrane distillation (MD) [1]. The concept of MD emerged in the 1960s, and was a topic for researchers exploring new membrane materials and systems with high thermal efficiency, but MD literature reported few full-scale installations. MD was previously limited by the availability of membranes, but with the recent availability of high-performance hydrophobic microfiltration membranes, MD may find practical application alongside the highly efficient reverse osmosis, and mature conventional thermal technologies (e.g., multi-stage flash). Furthermore, opportunities that appear suited to MD can be found due to its compactness as a thermal process and the low cost of materials. MD is a thermally driven separation process which uses a hydrophobic membrane as a physical barrier for polluted water, from which mass transport of vapor is driven by differences in vapor pressure [1]. Therefore, the critical feature of the membrane is its ability to resist pore wetting when in contact with the liquid to be treated [2]. Of the four main MD configurations, direct contact MD (DCMD) is the simplest and most studied for desalination and concentration of a range of industry solutions [3]. In this configuration, the heated solution (feed) is in contact with the surface of the membrane, and is designated the “hot side” where evaporation takes place at the feed–membrane interface. Water vapor is driven by the vapor pressure difference, caused by the temperature gradient across the membrane to the flowing, cold permeate side which condenses into the permeate stream. Because of the hydrophobic characteristic of the membrane, the liquid wastewater cannot penetrate the membrane. This configuration is known for having the highest fluxes, but the main drawback is the heat lost through conduction [3].

In general, the wastewater generated from the textile industry is high in chemical oxygen demand (COD), salt, and surfactants because it comprises spent textile dyes, suspended solids, mineral oils, electrolytes, surfactants, etc. [4]. All this makes textile wastewater difficult to treat.

Treatment of textile wastewaters was explored by several researchers [5,6,7,8], highlighting the limitation of conventional technologies. Traditional aerobic biological treatment is generally ineffective because of the poor biodegradability of dyes, and the inhibition of many biological processes [5]. Anaerobic or anoxic pre-treatments are required, as they are better suited for biodegradation of dyes. Coagulation is generally most effective with ferrous sulfate and a cationic polyelectrolyte [6], but it is often unsuitable for streams containing reactive or acid dyes because their removal is less efficient [5,7]. Ozonation can be effective for oxidizing many organic species, but it is ineffective against sulfur, vat, and disperse dyes [5]. Fenton oxidation using Fe^2+^ in the presence of hydrogen peroxide is generally more effective than ozonation, and can be operated at lower cost. Membrane techniques are effective at removing salts [8], but their performance is impacted by fouling from dyes. Moreover, the recovery is limited by the salinity. Adsorption via activated carbon or biomass is typically used, but its limited applicability, low commercial use, and complexity reduce its utility [7]. With wastewater so difficult to treat with one or even multiple processes, exploration of alternatives is needed, and there are few reports on the potential of MD in the treatment of textile wastewater.

Similar to other membrane technologies, MD faces two effects that are mainly responsible for the degradation of the membrane performance: fouling and wetting. The literature shows that fouling issues in MD cause severe flux decline, while wetting issues cause loss of the membrane hydrophobicity, leading to a passage for contaminants from the feed side into the permeate, and affecting the quality of the water produced [9,10,11]. The literature also shows that biological processes can remediate these issues by biologically removing retentate carbohydrates and proteins [10]. On the other hand, the membranes can be made to tolerate components that would otherwise compromise their performance. For example, researchers recently modified hydrophobic polyvinylidene fluoride (PVDF) membrane surfaces to create a hydrophilic surface that demonstrated resistance to fouling from oils in solution [12]. However, surfactants can also wet membranes, and membranes are yet unexplored for this application. It is already understood by the textile industry that resistance to surface wetting agents is provided by the addition of hydrophilic coatings. Hydrophilic-coated polyurethane is well known for application to breathable water repellent fabrics, where the polyurethane is coated on hydrophobic polytetrafluoroethylene (PTFE) to prevent contamination from oils and surfactants [13]. Indeed, the hydrophilic polyurethane coating is designed to be selectively permeable to water vapor. The underlying hydrophobic PTFE that prevents liquid water from migrating through the membrane is, therefore, protected by this coating. This commercial material is, therefore, an ideal candidate for the treatment of textile wastewaters rich in surfactants, and potentially fats and oils. The resistance to fats and oils by this membrane was demonstrated in previous work exploring the viability of MD applied in the meat industry [14].

This type of membrane may have multiple applications. Indeed, the literature shows that hydrophilic–hydrophobic membranes used with polyvinyl alcohol (PVA) coatings seemed highly efficient for the treatment of oily feeds via osmotic distillation [15]. In addition, they were successfully used in dispersion-free solvent extraction [16].

This work was concerned with the comparison of two PTFE membranes in the desalination of saline wastewater from a local textile factory operated by Australian Textile Mills (ATM), in an effort to make it suitable for internal re-use. This offsets the need for utilizing scarcer and higher-value potable water, while at the same time reducing the liquid disposal volumes.

This study follows on from two previous MD trials carried out by us. The first aimed to recover water from reverse osmosis (RO) concentrate of groundwater using solar thermal collectors [17]. The second aimed to recover water from ion-exchange regeneration waste produced at a power station, where the MD was powered by low-grade waste heat [18]. This paper presents the outcome of a pilot trial of MD for processing textile wastewaters, which was carried out before a three-month trial that was fed with wastewater from the existing biological treatment process [19]. However, direct treatment of the site’s effluent to avoid biological treatment aligns with the future vision of more intensified water treatment of textile facilities [20]. In moving toward this approach, the challenge is to prevent membrane wetting due to the presence of surfactants in the wastewater. State-of-the-art hydrophobic membranes, found to have high fluxes applied to desalination [21], were tested against a membrane through the novel use of a commercially available hydrophilic-coated PTFE membrane utilized in textile products. This membrane was fabricated for this work at the same textile facility.

## 2. Materials and Methods

### 2.1. Membrane and Module

Two different membranes were used to carry out this work. The first membrane selected demonstrated high performance in MD applications, and had an active layer made of polytetrafluoroethylene [21]. The PTFE active layer was supported by a scrim of polypropylene (PP) backing. The membrane was provided by Ningbo Changqi Porous Membrane Technology Co., Ltd. (Ningbo, China). The second membrane used in this work was a hydrophilic-coated hydrophobic PTFE membrane typically applied to waterproof breathable fabrics [13], and recently applied for MD [14]. The membrane was fabricated by ATM by adhering a woven support to the coated PTFE film to make the composite membrane.

The MD module used in this study was an in-house multi-layered design that was previously used in a power station trial [18]. Each membrane was hand cut and then placed between each divider, using gaskets and spacers for both sides. The use of turbulence-promoting spacers was used to improve flow uniformity and mixing in the channels [22], as the thickness of the thermal boundary layer can be reduced by improving the stream turbulence. The membranes used had an effective total area of 0.67 m^2^.

The MD module initially operated using the effluent taken from the company’s combined holding pit, where the wastewater exiting the processes is accumulated before being discharged. Due to the intermittent discharge of a secondary source of wastewater with a potentially high concentration of surfactants, the wastewater was collected from an intermediate tank drawn from the main effluent upstream from the combined holding pit.

### 2.2. Site Test Setup

The pilot plant was operated at the ATM site at Wangaratta, Victoria, Australia. The aims for the pilot plant were to treat the wastewater produced during the fabrics manufacture process, to produce water to a high-quality standard suitable for plant re-use, and to demonstrate the capability for using waste heat as the source of energy.

**Figure 1:**
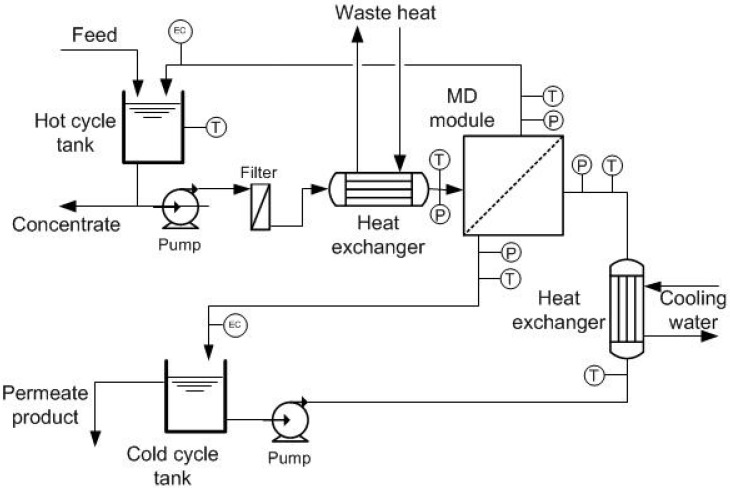
Membrane distillation (MD) pilot-scale system.

The plant was designed with an estimated capacity on the order of 480 L·day^−1^ based on the upper flux value of 30 L·m^−2^·h^−1^. However, flux (and plant capacity) is dependent on temperature, available waste heat, and membrane fouling. Stated flux and capacity assumed a 60-°C waste-heat source with approximately 14 kW of available heat, with cooling water at approximately 20 °C. The schematic of the MD pilot-scale system is shown in Figure 1.

The system consisted of two tanks with a capacity of 85 L each, which served as feed (hot loop) and permeate (cold loop) reservoirs. The hot loop had two filters (25 µm and 5 µm) used to reduce the amount of solids in the feed as the water became concentrated, thus reducing membrane fouling [23,24]. A plate heat exchanger (HX) was used for the heat transfer between the feed and the heat source. The heat source was provided from the site’s steam-condensate return line to the boilers. The temperature of this condensate was highly variable, fluctuating between 80 °C and 100 °C. The permeate was cooled in a plate heat exchanger using the same site’s cooling water circuit (15–20 °C). Both the hot feed and the cold permeate loops were pumped by two centrifugal pumps in a counter-flow arrangement through the membrane module. The plant was equipped with control systems and data logging to enable continuous unattended operation.

The temperature and pressure of the feed and permeate were monitored at their respective inlets and outlets using data logging. Electric conductivity (EC) sensors were used in the feed and permeate cycles to indicate the concentration of dissolved solids, which was translated to salt rejection. Cycle flow rates on both sides were adjusted using pressure regulators placed at their respective entrances to the membrane module. The typical flow was around 16 L·min^−1^ in the feed loop, and 14 L·min^−1^ in the permeate loop, controlled by the set module inlet-gauge pressure of 20–35 kPa on each side. Sodium sulfate solution (30 g·L^−1^) was used as feed at the beginning of each test to confirm the membrane performance flux and salt rejection. Upon starting operation of the MD plant, evidence of intact membranes and their effective operation was indicated by the permeate tank increasing in level while EC declined. Meanwhile, the feed tank decreased in level and increased in EC.

### 2.3. Water Quality Analyses

Analyses of the effluent and the permeate produced were carried out to indicate the separation performance of the system and membrane intactness. Various parameters were measured to determinate their quality. The total dissolved solids (TDS) was estimated using the EC value. The conversion factor, dependent on the chemical composition of the TDS, can vary between 0.55 and 0.8. A value of 0.63 is commonly used as an approximation if the actual factor is not known [25] (TDS ≅ 0.63 × EC). The EC and the pH were measured using a WTW Multiline P3 pH/LF equipped with an EC meter and a pH probe. The values of total nitrogen (TN), nitrate (NO_3_^−^), nitrite (NO_2_^−^), ammonium (NH_4_^+^), chemical oxygen demand (COD), and total phosphorus (TP) were obtained using Merck Millipore pre-prepared test kits for the Spectroquant instrumentation: 1.14763.0001 (TN), 1.14547.0001 (NO_2_^−^), 1.14542 (NO_3_^−^), 1.14543.0001 (TP), 1.14558.0001 (NH_4_^+^), 1.14541.0001 (COD), and Merck Millipore Spectroquant Pharo 300.

The rejection of the compounds was defined by the membrane rejection coefficient, defined as

(1) $$R=1-\frac{C_{f}}{C_{r}},$$

where *R* is the rejection of the membrane for a given component; *C_f_* and *C_r_* are the concentrations of the rejected component in the filtrate (permeate) and the retentate, respectively [26].

Finally, the flux through the membrane (*J*) was determined as

(2) $$J=\frac{Qp}{A}.$$

Interpreted as the process productivity, it was defined by the permeation rate, *Qp*, divided by the membrane surface, *A*. It also represented the velocity of the fluid perpendicular to the surface of the membrane [27].

### 2.4. Scanning Electron Microscopy

Scanning electron microscopy (SEM) images were obtained using a Nikon/JEOL Neo-Scope JCM-5000 SEM (Melville, NY, USA) with 10 kV accelerating voltage and capturing secondary electron detection. All specimens were dried in a desiccator overnight, and gold-coated for two minutes before SEM analysis.

## 3. Results and Discussion

### 3.1. Membrane Analysis

**Figure 2:**
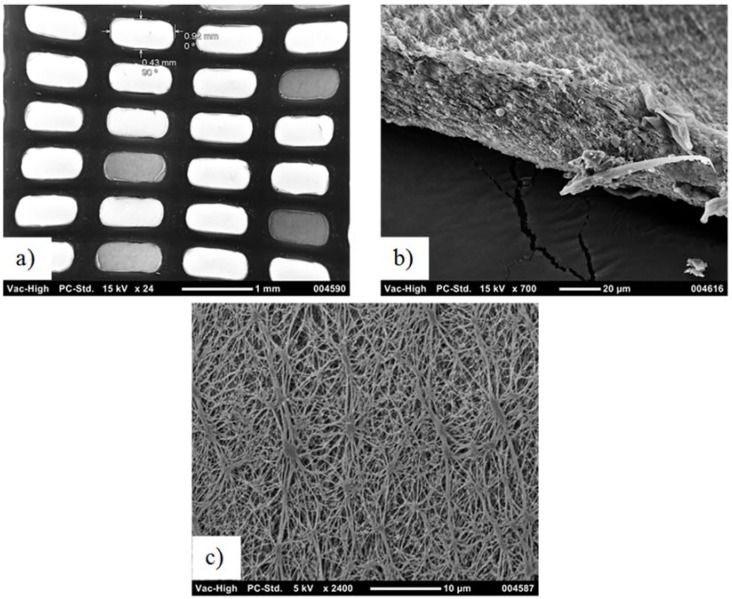
Standard polytetrafluoroethylene (PTFE) membrane: (**a**) scrim support (backing); (**b**) membrane side view; and (**c**) membrane front view.

**Figure 3:**
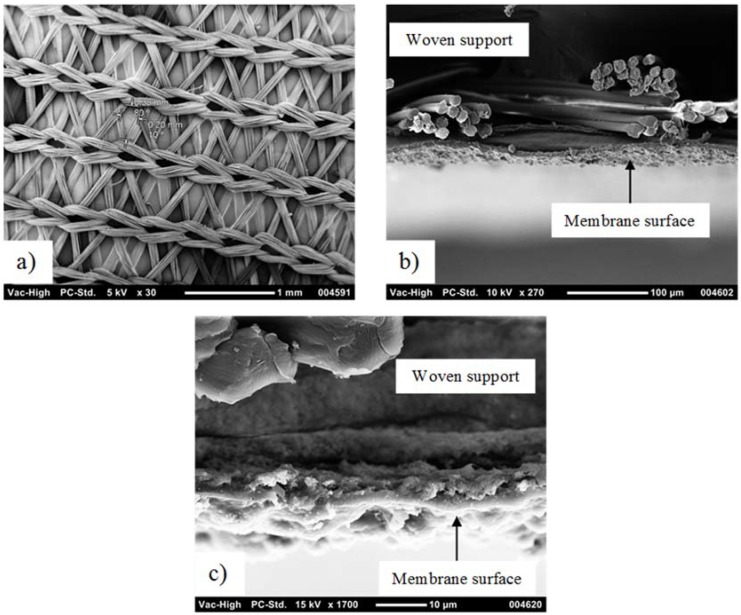
Hydrophilic-coated hydrophobic PTFE membrane: (**a**) woven support (backing); (**b**) side view; and (**c**) side view (zoomed in).

Figure 2 and Figure 3 show the SEM images of the two membranes utilized in this study. The left (a) images show the membrane from the support side, and the center images show the membrane cross-section. Figure 2c shows the PTFE surface of the standard hydrophobic membrane, while Figure 3c shows a closer view of the cross-section of the coated PTFE. The coating appears dense from SEM analysis, as previously reported for the same membrane type [14]. Comparing Figure 2 with Figure 3 shows greater membrane exposure for the scrim-supported membrane, leading to the conclusion that the scrim support with wide openings used on the standard PTFE membrane was expected to lead to higher flux than the tighter-woven support used on the hydrophilic-coated hydrophobic PTFE membrane. Also, pores on the standard hydrophobic PTFE membrane can be seen in Figure 2c, while, as reported previously, the coated membrane appeared similar when imaging the PTFE through the support while the polyurethane coating was dense [14]. The membranes had the key elements for application to an MD pilot module (supported thin porous hydrophobic layers).

### 3.2. Initial Plant Performance

**Figure 4:**
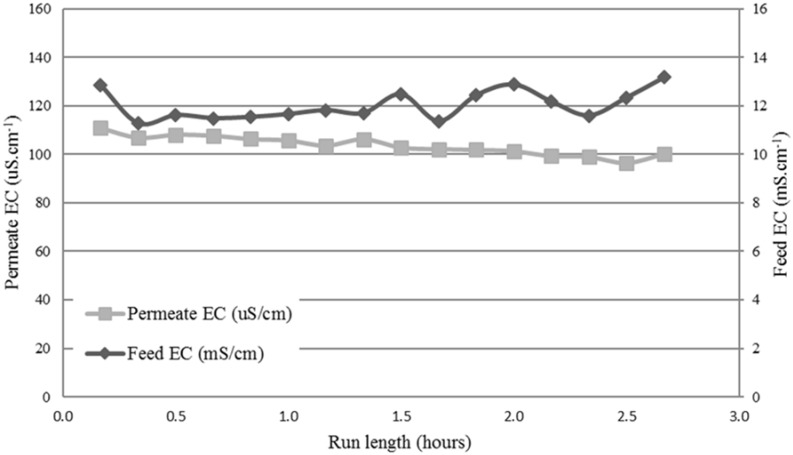
Initial performance of the MD pilot using Na_2_SO_4_ to verify the intactness of the standard PTFE membrane. Results show the electrical conductivity (EC) of both the feed and permeate tanks.

Figure 4 shows the typical results obtained with the Na_2_SO_4_ test on the standard PTFE membrane. The Na_2_SO_4_ solution was used as a means to check pilot plant performance before adding the textile wastewater. The aim of this was also to confirm the intactness of the membrane module sealing. The wastewater feed was then added after flushing the membrane and rig with clean tap water.

The EC slowly rose on the feed side while it decreased slowly on the permeate side. A salt rejection of 99.5% was calculated from the EC values. The membrane intactness was confirmed by this test, and could now receive wastewater from the site. The same test was carried out on the hydrophilic-coated hydrophobic PTFE membrane, obtaining similar results.

### 3.3. Analysis of Wastewater Used in Trial

The feed effluent (wastewater) quality was highly variable due to several production operations occurring intermittently. However, Table 1 shows typical operational values, and those representing the tests presented here.

The wastewater was relatively high in COD, as expected for textile wastewaters [4,28]. The COD represents the organic material in the water, which is expected to cause serious fouling of the membranes. The pH value was in the range estimated for this kind of effluent [29]. The EC and TDS of the wastewater were moderate, but depended on the processes used at the site. In this case, the EC and TDS values were slightly lower than expected. NO_3_^−^ values were especially low for textile wastewater. Typical values were over 100 mg·L^−1^ [30]. In textile effluents, ammonia can be present in large quantities. In this case, the values were lower than reported in previous literature [31]. Ammonia can evolve into the permeate in MD due to its volatility. Furthermore, some works showed that the pH of the feed had a significant effect on the rate of the removal efficiency of easily ionized volatile compounds, such as ammonia [32].

### 3.4. Performance of Standard Hydrophobic PTFE Membrane

**Figure 5:**
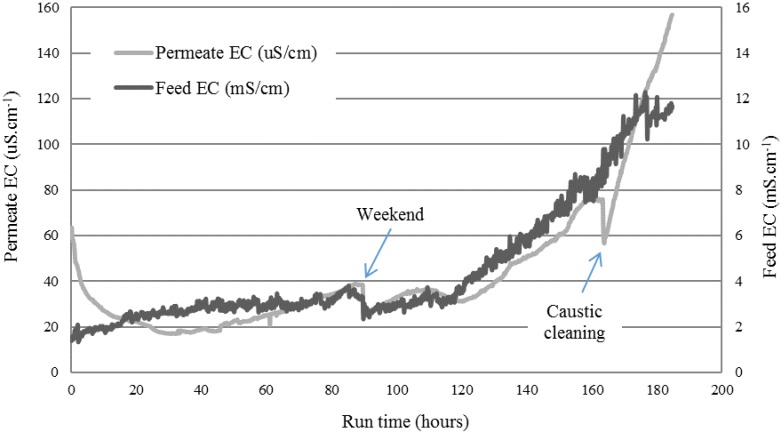
EC of the feed and permeate cycle containers while treating the textile wastewater on the hydrophobic membrane.

Figure 5 shows the feed and permeate profile. The average flux over the course of this test was 12 L·m^−2^·h^−1^. The trial was operated over 10 days, corresponding to a runtime of 190 h. Over this time, the feed was concentrated from 2 mS·cm^−1^ to around 12 mS·cm^−1^, suggesting about a six-fold concentration ratio. Two different profiles of rising EC can be seen on the feed side. Initially, in the first 120 h of operation, the EC increased slowly at a rate of 0.012 mS·cm^−1^·h^−1^. After this time, a 10-fold increase in a linear profile (R^2^ = 0.9647) was observed between 120 h and 160 h (0.133 mS·cm^−1^·h^−1^). Meanwhile, permeate EC dropped in the initial 10 h, showing that higher-quality distillate emerged through the membrane into the permeate cycle, which was initially filled with tap water (tap water EC ranges between 80 μS·cm^−1^ and 100 μS·cm^−1^). However, the EC started rising slowly between 40 h and 80 h. After the weekend break, the EC fluctuated, but started rising at 120 h. This rise was linear (R^2^ = 0.9815) with a rate of 1.14 μS·cm^−1^·h^−1^ until 160 h. At this stage, caustic cleaning (1 wt % NaOH at room temperature ~26 °C) took place, which initially led to a slight reduction in the permeate EC, but EC then immediately started rising again at a much faster rate of 4.2 μS·cm^−1^·h^−1^.

Noting the rate of increase in EC, it is indicative of membrane wetting. The rapid increase in the permeate EC suggests liquid transfer from the feed into the permeate through wetted membrane pores. This was confirmed by the observation of foam and color in the permeate tank after 120 h of the run. One important aspect that resulted from this experience is the possible contribution of sodium hydroxide cleaning to the wetting. Saponification reactions of organics with sodium hydroxide in the solution are a possible reason, as these lead to surfactants, which lead to membrane wetting. In fact, oil and greases are typically found in textile wastewaters [31]. This oil could then stick to the membrane, creating a saponification reaction when reacted with NaOH solution. This effect, together with the gradual wetting observed during the run, was the main motivation for testing a membrane that contained a hydrophilic coating, in an attempt to avoid the wetting effect from surfactant by-products from membrane cleaning.

**Figure 6:**
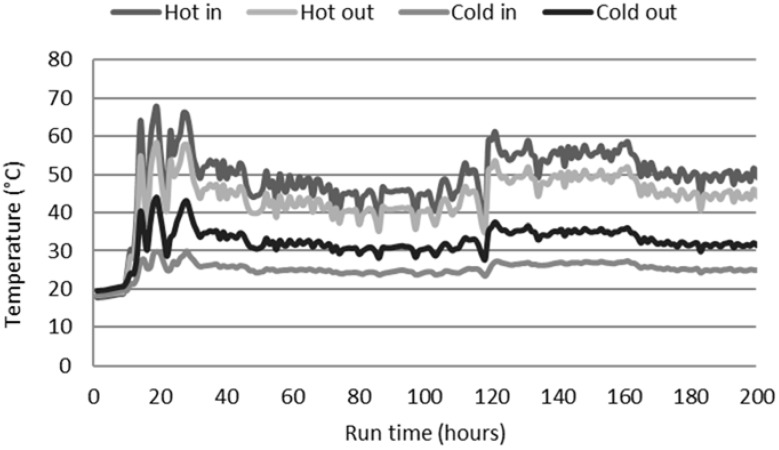
Temperature profile using the standard hydrophobic membrane.

Figure 6 shows the temperature profile obtained using the standard hydrophobic membrane. The feed side “hot in” temperatures ranged between 35 °C and 60 °C, representing the temperatures available from the waste heat at this site.

Table 2 shows the water quality indicators of the retentate (concentrated waste) and permeate just before the cleaning operation. The permeate product was of good quality, where EC, nitrate and nitrite, COD, and TP all exhibited more than 98% rejections across the membrane.

A salt rejection of 98.9% based on EC was demonstrated during the majority of the run, which was expected for this membrane and system [17,18]. However, rejection reduced by 0.6% (absolute) when compared with the Na_2_SO_4_ test. High-temperature fluctuations on the feed side, as shown in Figure 6, affected the constant production of permeate. The feed temperature seemed to be key in the water recovery. High temperatures increased the production as a result of increased vapor pressure [4,33]. However, an increase in the feed temperature could potentially increase the fouling due to precipitation of negatively soluble salts, such as calcium salts, via scaling [34]. Tests showed that the introduction of a filter just after the hot loop HX reduced the deposition of bulk organic or colloidal matter on the membrane much like it improved water recovery, achieved via removing the calcium sulfate which scaled the membrane, as shown in previous work [24]. Visual observation of the membranes to determine fouling will be shown later in this section.

The salt rejection of almost 99% estimated from EC meant that the permeate produced was even lower in salts than normal tap water determinate at 100 µS·cm^−1^. A COD rejection of 98% indicated a high level of rejection of organic matter in the feed. TN, NO_3_^−^, NO_2_^−^, and TP had a high level of removal (over 97%). The lower (64%) rejection for TN when compared with that of COD or EC was potentially due to ammonia. Due to its volatile nature, ammonia can pass through the membrane as vapor, and condense in the permeate [35], thereby contributing to the increase of the permeate EC values. However, in this case, EC rejection remained very high, close to the nonvolatile species represented by TP, suggesting that the majority of the ammonia was in nonvolatile ammonium form (or organically bound), and remained on the feed side of the membrane. This was due to carbonate being required for it to pass through the membrane [36], which was not likely present in the raw site effluent. The feed pH of 7.8 suggested that the majority of the ammonia was in the nonvolatile ammonium form.

**Figure 7:**
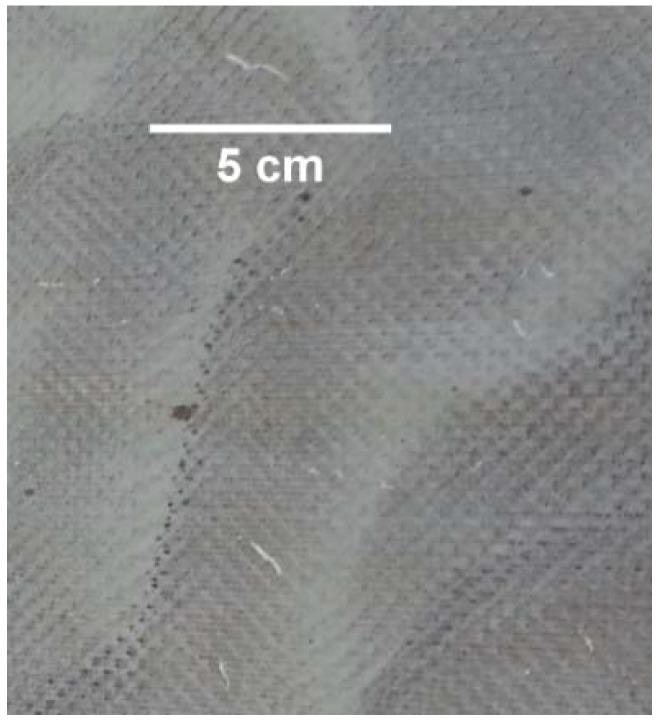
Image of hydrophobic PTFE membrane after treatment of textile wastewater.

Due to the concentrating effect of the feed solution, inorganic and organic species build up, causing fouling and wetting. Figure 7 shows the appearance of the membrane after the run. The darker regions are indicative of wetting, as they are actually transparent, known to indicate where the membrane is wetted [19]. Therefore, MD is likely to require pre-treatment or inline removal of accumulated solids to maintain performance and produce high-quality water. This observation was in agreement with previous work [24,28,34]; the use of pre-treatment is highly recommended for decreasing the concentration of organics and reducing the presence of foulants. The use of pretreatments, such as flocculants or foam fractionators (used to remove organic and inorganic compounds via a rising stream of fine air bubbles), are recommended for further studies, as it might be a good improvement to reduce the level of surfactants in the feed wastewater before it is introduced to the MD plant. In a recent trial paper [19], the use of fractionation was demonstrated at bench scale to minimize surfactant wetting. The combination of processes, such as a membrane bioreactor (MBR) or nanofiltration/ultrafiltration (NF/UF), with MD is likely an option [20].

### 3.5. Performance of Hydrophilic-Coated Hydrophobic PTFE Membrane

**Figure 8:**
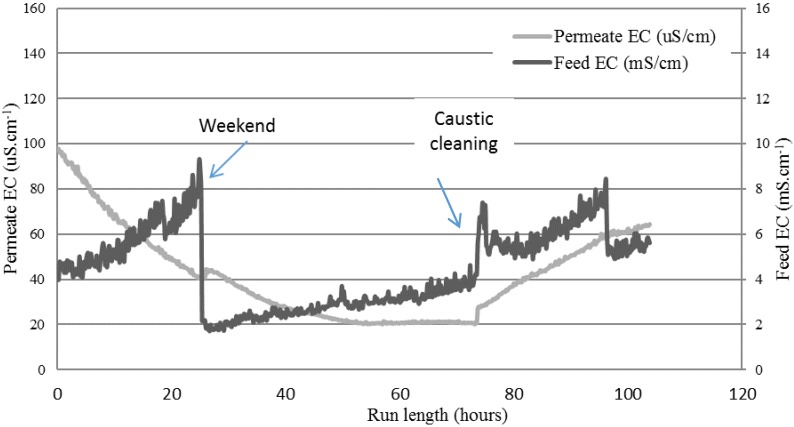
EC of the feed and permeate cycle containers while treating the textile wastewater on the hydrophilic-coated hydrophobic PTFE membrane.

The profile of the EC using the coated membrane for treating the textile wastewater is shown in Figure 8. This trial was operated over seven days, corresponding to a total runtime of 106 h. Over the first 25 operating hours, the feed was concentrated from 4 mS·cm^−1^ to around 7.5 mS·cm^−1^, suggesting about a two-fold concentration ratio. Flux at 20 h was 5.2 L·m^−2^·h^−1^. After the weekend shutdown, it increased to 6 L·m^−2^·h^−1^, decreasing to 4.8 L·m^−2^·h^−1^ before cleaning, and then increased to 5.2 L·m^−2^·h^−1^ after cleaning. At the end of the run, flux reached 4 L·m^−2^·h^−1^. After the weekend, new feed effluent was added into the feed tank, which explains the sudden EC drop. A different trend of rising EC was observed on the feed side. Initially, in the first 25 h of operation, the EC increased significantly at a rate of 0.15 mS·cm^−1^·h^−1^. After the weekend, the rising was an order of magnitude slower (0.03 mS·cm^−1^·h^−1^) just before the caustic cleaning. On the permeate side, EC dropped until the caustic cleaning (about 73 operating hours), showing a higher-quality distillate, with EC values lower than those typical of tap water. However, the EC started rising slowly after the cleaning.

The salt rejection rate exceeded 98% based on EC, demonstrating a high degree of salt rejection. Moreover, the quality of the permeate was better than normal tap water, with EC values around 40 µS·m^−1^ just before the weekend shut down. After the weekend, the EC in the permeate continued decreasing until it reached 20 µS·cm^−1^, where it stabilized.

The hydrophobic PTFE membrane showed higher average fluxes (~12 L·m^−2^·h^−1^) than the coated PTFE membrane (~5 L·m^−2^·h^−1^) when processing the textile wastewater. The differences in fluxes could be due to either the coating reducing the mass transfer, thereby lowering the flux, or different features of the scrim layer inhibiting mixing on the permeate side, or the different PTFE membrane properties (pore size, porosity, or thickness). The hydrophilic-coated hydrophobic membrane varied according to these three conditions in comparison to the hydrophobic PTFE membrane, as shown in Figure 2 and Figure 3. Optimizing the performance of this membrane for MD application is subject to further work in this study. However, this study aimed to first verify if the new membrane chemistry would resist the wetting nature of the real textile industry wastewater when treated by MD. A key difference observed here was the ongoing decline in the permeate EC, where the hydrophobic membrane only showed a declining EC in the first 30 h of operation (Figure 5), before rising EC occurred from then on, starting when the feed EC was only around 3 mS·cm^−1^. Meanwhile, the coated PTFE membrane showed a continual decline for more than 70 h of exposure to the wastewater, where feed EC peaked at around 9 mS·cm^−1^. The coated membrane was, therefore, able to resist the wetting experienced by the hydrophobic PTFE membrane.

**Figure 9:**
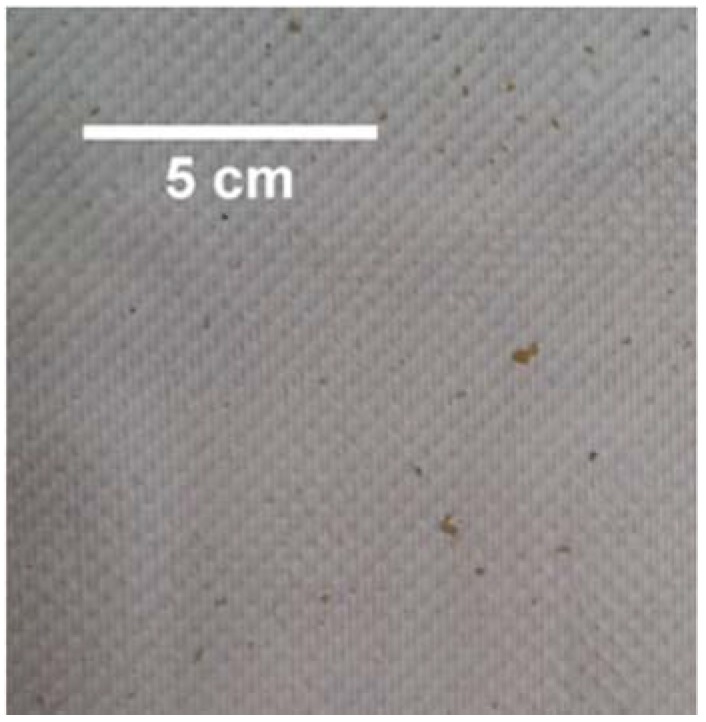
Image of the hydrophilic-coated hydrophobic PTFE membrane after testing with textile wastewater.

Cleaning of the coated PTFE membrane was also investigated. A sodium hydroxide 1 wt % in-place clean was carried out at room temperature. The feed EC rose and fell, as shown in Figure 8 between 73 h and 76 h, and was probably due to the time required for stabilization following membrane cleaning and flushing. As with the cleaning of the hydrophobic PTFE membrane, a rise in permeate EC was also noted here. Permeate EC began rising after the clean at 1.44 μS·cm^−1^·h^−1^, which, when compared with the 4.2 μS·cm^−1^·h^−1^ from the hydrophobic membrane, shows potentially better handling of the in-place cleaning process. Although the coated PTFE membrane was superior to the hydrophobic PTFE membrane in regular operation, and resisted the wetting effect more strongly, cleaning compromised its integrity as a salt-rejecting membrane [37]. Figure 9 shows the membrane appearance after treatment of the textile wastewater (including the cleaning operation). Some spots appeared on the surface, which were likely due to fouling from larger particles. However, the coated membrane showed no obvious wetting (transparent areas) when compared with the hydrophobic membrane (Figure 7). Regardless, sodium hydroxide cleaning affected both membranes. While this is useful in cleaning membranes, as the sodium hydroxide can increase the solubility of solutes via hydrolysis and solubilization [38], wetting by-products are an issue for MD membranes. Therefore, further work on coated membrane types is needed to confirm its ability to clean membranes without compromising their underlying hydrophobic property.

Table 3 shows the quality of the permeate produced and the retentate. The permeate EC value was slightly lower when compared with the hydrophobic membrane, proving that although both membranes had a high salt-rejection capacity, the coated membrane was superior for operation in the highly wetting effluent when compared with the standard hydrophobic type. A COD rejection of 97% was achieved, while TN and NO_2_^−^ had a high level of rejection (over 98%). Ammonia levels were slightly lower in this feed, but transfer to the permeate was still evident from the lower rejection when compared with nonvolatile components such as phosphorus, indicated by TP. TDS rejection was achieved, based on an EC of 99%. The values of NO_3_^−^ and TP, which were higher than typical, verified the variability of the effluent. Textile effluent quality is highly variable, and hard to predict. It is unknown if the rising EC after cleaning was due to wetting, as the feed EC was also rising; an EC rise on the feed side can also lead to rising permeate EC. However, as permeate EC was not rising rapidly, the membrane was still potentially intact. Nevertheless, the probability of some degree of loss of membrane integrity is high. Therefore, this membrane may be a robust material for MD applications of textile effluents, but further work is needed to explore the effects of cleaners and their impact over time.

**Figure 10:**
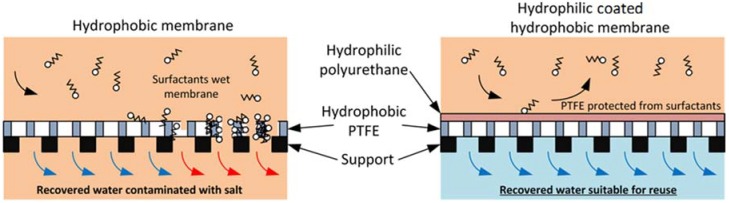
Figure explaining wetting on both membranes.

Figure 10 summarizes the wetting phenomenon on both membranes.

## 4. Conclusions

This study aimed to compare standard hydrophobic PTFE membranes used for MD with a novel hydrophilic-coated PTFE membrane material for the treatment of textile industry wastewater. Uniquely in this work, the approach of the trial was the treatment of effluent directly from the plant, for a future vision of intensified membrane processing of wastewater when compared with the current use of biological treatments. Therefore, wetting from surfactants was an expected problem, which was a focus of the application of the coated PTFE membrane used. The MD process needed to demonstrate its viability to produce high-quality water suitable for plant re-use, utilizing real plant temperature from available waste heat.

The standard hydrophobic PTFE membrane demonstration showed a good salt-rejection rate of around 99%. However, gradual wetting was observed, indicated by a rise in EC after 30 h of operation. Ammonia transport into the permeate, which can influence EC measurement even on intact membranes, was ruled out, as the permeate started visibly foaming. Furthermore, observation of the membrane removed from the rig showed transparent spots on the surface, identifying wetted areas. The coated PTFE membrane, on the other hand, exhibited superior wetting resistance. However, both membranes showed a rising EC after cleaning with 1 wt % NaOH solution at room temperature, which was attributed to wetting of the membranes. The coated membrane’s anti-wetting performance under conventional operation was demonstrated, but further developments in cleaning methods are needed for its sustainable operation.

## Figures and Tables

**Table 1:** Typical feed effluent quality.

Parameter	Value
pH	7.76
Electrical Conductivity (EC; µS·cm^−1^)	976
Total Dissolved Solids (TDS; mg·L^−1^)	605
Total Nitrogen (TN; mg·L^−1^)	11
Nitrate (NO_3_^−^; mg·L^−1^)	8
Nitrite (NO_2_^−^; mg·L^−1^)	0.97
Ammonium (NH_4_^+^) (mg·L^−1^)	1.12
Chemical Oxygen Demand (COD; mg·L^−1^)	2830
Total Phosphorus (TP; mg·L^−1^)	3.10
Color	Colored

**Table 2:** Retentate and permeate quality of the standard hydrophobic membrane.

Parameter	Retentate (before Cleaning)	Permeate (before Cleaning)	Rejection (%)
pH	7.8	6.25	-
EC (µS·cm^−1^)	3220	34.2	98.9
TDS (mg·L^−1^)	1250	14.9	98.8
TN (mg·L^−1^)	21	0.6	97.1
NO_3_^−^ (mg·L^−1^)	8	0	100.0
NO_2_^−^ (mg·L^−1^)	2.23	0.02	99.1
NH_4_^+^ (mg·L^−1^)	1.67	0.6	64.1
COD (mg·L^−1^)	3350	61	98.2
TP (mg·L^−1^)	4	0.06	98.5

**Table 3:** Retentate and permeate quality of the hydrophilic-coated hydrophobic PTFE membrane.

Parameter	Retentate (before Cleaning)	Permeate (before Cleaning)	Rejection (%)
pH	6.21	7.86	-
EC (µS·cm^−1^)	3650	19	99.5
TDS (mg·L^−1^)	2300	11.97	99.5
TN (mg·L^−1^)	42	0.9	97.8
NO_3_^−^ (mg·L^−1^)	>18	13.5	25
NO_2_^−^ (mg·L^−1^)	0.749	0.025	96.7
NH_4_^+^ (mg·L^−1^)	1	0.4	60
COD (mg·L^−1^)	4335	284	93.4
TP (mg·L^−1^)	15.2	0.5	96.7
